# The Development and Implementation of a Simulation Orientation Curriculum for Newly Hired Pediatric Emergency Medicine Attending Physicians

**DOI:** 10.1002/aet2.70138

**Published:** 2026-02-18

**Authors:** Michael Hrdy, Megan Lavoie, Marleny Franco, Grace Good, Theresa Walls, Khoon‐Yen Tay

**Affiliations:** ^1^ Division of Emergency Medicine Children's Hospital of Philadelphia Philadelphia Pennsylvania USA; ^2^ Department of Clinical Pediatrics University of Pennsylvania Perelman School of Medicine Philadelphia Pennsylvania USA; ^3^ Center for Simulation, Advanced Education and Innovation, Children's Hospital of Philadelphia Philadelphia Pennsylvania USA

## Abstract

**Background:**

The transition from trainee to new attending physician can be overwhelming. Simulation has been shown to be effective in orienting trainees to the emergency department (ED) but there is limited literature on simulation for the orientation of newly hired attending physicians.

**Objective of the Innovation:**

The objective of this innovation was to develop, implement, and evaluate a simulation curriculum to supplement the orientation of newly hired attending physicians in our emergency department.

**Development Process and Implementation:**

We developed a year‐long quarterly simulation curriculum guided by Kern's six‐step curricular design model. Based on a targeted needs assessment, nine scenarios were chosen for inclusion. These scenarios were arranged to start the curriculum with those simulation scenarios that highlight systems and processes particular to our institution, with a transition towards scenarios requiring more complex team leadership and medical management decisions in the latter portions of the curriculum.

**Outcomes:**

We have completed two years of this curriculum and have had 14 participants in total. Thirteen participants completed the end‐of‐year evaluation. The curriculum has been well received by participants with unanimous agreement that the curriculum helped them lead acute patient scenarios in the resuscitation bay during their first year as a new hire.

**Conclusions:**

Simulation for the orientation of new attending physicians can be implemented successfully and received well by the targeted learners and by leadership invested in supporting new attending physicians. While the specific scenario topics and institutional procedures are site‐specific, the approach to curricular design and implementation is widely generalizable to other EDs.

## 1 Need for Innovation/Background

The transition from trainee to attending physician can be overwhelming. A new attending physician is faced with challenges including role identity formation, task completion, and leadership of a clinical team [1]. Not only are attending physicians in a new clinical role, they are often also in a different hospital system from their training. New attending physicians in emergency medicine are faced with a particularly difficult set of leadership challenges given the interdisciplinary nature of the emergency department team, the variability of patients presenting on shift, and the need for rapid, high‐stakes decision making [2, 3, 4]. In recognition of these challenges, consensus recommendations have been developed to ease the transition from emergency medicine resident to attending physician [5].

In agreement with these recommendations, many academic emergency departments (EDs) have recognized the stress inherent in this transition and have implemented a more formal orientation process to the attending role. The benefits of these orientations have been demonstrated in the literature [6, 7]. These orientations tend to be didactic or asynchronous in nature and involve the dissemination of logistical information, the establishment of faculty expectations, and the initiation of a culture around mentoring rather than providing supervised in situ practice of clinical skills and team leadership.

Simulation has been used in various medical settings and with varied experience levels of learners to aid transition to new roles. Studies have shown that for medical students, nurses, and resident trainees in both surgical and nonsurgical specialties, simulation has led to improvements in comfort and competency, as well as sustained improvements in procedural skills when used in the orientation setting [8, 9]. In our experience, and in the literature, there is little that exists regarding simulation programs that have been developed to clinically orient new emergency medicine attending physicians in a hands‐on manner despite simulation being a frequent component of orientation to the emergency department for emergency medicine and pediatrics residents and simulation being shown to be helpful for continuing education for emergency medicine physicians [10, 11, 12, 13, 14, 15, 16, 17, 18].

Simultaneously, the culture in many institutions is such that attending physician development and continued education take a back seat to trainee education. Given this, we recognized a need for, and then created, a clinically‐oriented in situ simulation curriculum for newly hired pediatric emergency medicine (PEM) attending physicians in our ED.

## 2 Objective of Innovation

**FIGURE 1:**
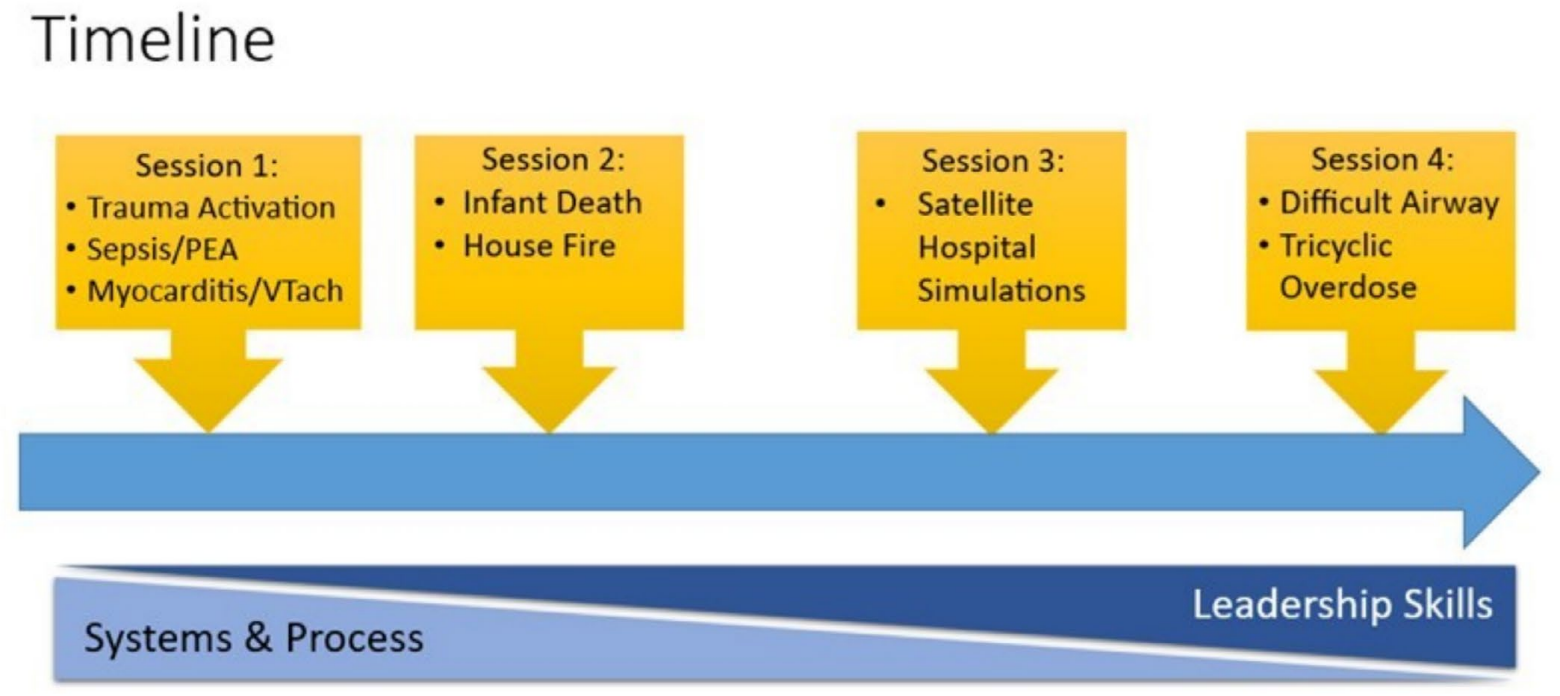
Simulation curriculum for attending physician orientation overview. Over the course of the curriculum, the emphasis of the scenarios shifted from orientation to institution‐specific systems and processes toward a focus on leadership skill development.

The objective of this innovation was to develop, implement, and evaluate a simulation curriculum to supplement the orientation of newly hired attending physicians in our ED. Our aim was to start the curriculum with simulation scenarios that highlight systems and processes particular to our institution with a transition towards scenarios requiring more complex team leadership and medical management decisions in the latter portions of the curriculum (Figure 1). The target audience of this curriculum was pediatric emergency medicine faculty attendings in their first year after being hired, regardless of whether the attendings had prior clinical experience in our institution or whether they were recent graduates of a training program. The results of this innovation may inform the creation of simulation curricula for the orientation of new attending physicians in pediatric and general emergency departments.

## 3 Development Process

We developed a year‐long quarterly simulation curriculum guided by Kern's six‐step curricular design model [19]. After identifying the problem of needing to orient new faculty to the complex environment of our quaternary care hospital's ED (Step 1), we performed a targeted needs assessment to identify the needs of our new attending physicians (Step 2). We surveyed attending physicians that had been hired in the previous two years about their perceived need for a simulation orientation curriculum. The needs assessment included questions about which clinical scenarios they felt would be helpful to include as well as the ideal timeframe in which the simulations should be performed. Based on this data, we identified learning objectives (Step 3) and constructed simulation scenarios that would meet those objectives (Step 4).

Simulation was chosen as the educational modality to address this gap in attending physician orientation based on the ability of simulation to educate along the entirety of Kolb's experiential learning cycle as well as the ability of simulation education to be tailored to learners' individual needs, respecting adult learning principles [20, 21, 22]. After reviewing our targeted needs assessment and proposed simulation curriculum, divisional leadership was supportive of a mandate that new faculty attend the simulation sessions to gain more immersive experiences in our acute clinical spaces over their first year. New attending physicians are not scheduled for clinical work for the 24‐h period surrounding a curricular session to ensure they can engage and participate fully.

Participants were surveyed after the first session of the year as well as at the end of the year to evaluate their impressions of the curriculum and identify any potential changes required to the curriculum.

## 4 The Implementation Phase

Thirteen potential scenario topics (see Appendix S1) were generated by our group based on our collective experiences at our institution and included in the needs assessment. From this list, recently hired attending physicians were asked to determine whether they thought new attending physicians should be exposed to the scenarios within the first 3, 6, or 12 months of their first year. Suggestions for additional topics not included in the initial list were also solicited. Based on the results of the needs assessment, septic shock with progression to PEA arrest, level 2 trauma upgraded to level 1 trauma, and ventricular tachycardia requiring synchronized cardioversion were included in the first session with new faculty. This initial 3‐h session was scheduled for September, which was within 3 months of all of the new attending physicians' starting dates. These scenarios were chosen because of the complex and unique ED systems and processes or specifically used equipment (e.g., defibrillator) that are often utilized in these scenarios to help orient new attending physicians to the nuanced institutional policies/procedures surrounding these high‐risk scenarios.

The scenarios occurred in the clinical environment and we recruited an interdisciplinary team including nurses and respiratory therapists (RT) to participate in the simulation sessions to enhance the fidelity of the scenario and to help achieve teamwork, leadership, and communication objectives. Nurses were primarily recruited from individuals who were working clinically that the charge nurse provided clinical coverage for. RT participation was facilitated either through recruiting individuals working clinically or having a lead RT attend. The bootcamp scenarios were facilitated by a team of PEM‐simulation faculty and were structured in a reflection‐on‐action format with frequent short breaks at critical moments in the scenario to allow for discussion, questions, and clarification [23].

The remaining scenarios were spread out in quarterly sessions over the course of the academic year. These sessions were each one hour and forty‐five minutes in length and covered two simulation scenarios. The participant group was divided in half and the sessions were run twice so that the learner groups were smaller and each individual new attending physician could have ample opportunities to be the team leader.

In the first few years, all newly hired attending physicians were hired to work at our main campus plus the same satellite hospital ED; therefore, one of the quarterly sessions occurred at our satellite hospital ED and included scenarios that were specifically chosen to highlight nuances in systems and processes at the satellite that differed significantly from those at the main campus, namely bleeding after tonsillectomy (included for two years) and status epilepticus and ventriculoperitoneal shunt failure (included one year each) (Figure 1).

## 5 Outcomes

To date, we have completed two full cycles of the curriculum for 14 total new attending physicians (6 in the first cycle and 8 in the second). Attendance at the first session of each cycle was robust, with 100% (14/14) of new faculty participating. Participants were only required to attend the first session due to the hospital‐specific policies and systems being simulated. Individually, new faculty attended either 1 or 3 of the three remaining quarterly sessions, with 50%–100% present at each quarterly session. The participants who only attended one session (*n* = 4) had at least one year working as attendings before being hired.

Ninety‐three percent of participants (13/14) completed an evaluation after the first session of the year and 93% of participants (13/14) completed evaluations at the end of the year. Participants highlighted the benefit of opportunities to discuss how certain scenarios play out in our institution, to pause during scenarios to review intricacies of our systems, and to make mistakes in a safe space with veteran attending physicians as the most valuable aspects of their experience. When asked at the end of the curriculum if the simulation orientation curriculum was helpful for their experiences leading in the resuscitation bay, responses were uniformly positive, with 6/13 agreeing and 7/13 strongly agreeing.

When asked about the timing of scenarios, most participants felt that septic shock with progression to PEA arrest and level 2 trauma upgraded to level 1 trauma should be included within the first 3 months (66% and 83%, respectively). However, only 42% felt that ventricular tachycardia requiring synchronized cardioversion should be included within the first 3 months. Of the remaining scenarios, 66% felt that the difficult airway team activation scenario should have been earlier in the curriculum.

When evaluating the first session, 50% (7/14) of respondents mentioned that they felt the scenarios chosen were one of the most useful parts of the session. Forty‐two percent (6/14) of respondents mentioned the value contributed by being in the actual clinical environment, while 64% (9/14) appreciated the systems and process focus of the first scenarios chosen. The reflection‐on‐action format was highlighted in 57% (8/14) of the evaluations as being of value as well.

## 6 Reflective Discussion

After two years of feedback from new attending physicians participating in our curriculum, we feel that this approach to orientation for new faculty can be implemented successfully and received well by the targeted learners and by leadership invested in supporting its new attending physicians. The ability of simulation to engage across the entire experiential learning cycle was instrumental in the success and buy‐in of our orientation curriculum [20].

The details of our orientation curriculum are specific to our large academic pediatric emergency department (and the inclusion of those specifics was felt to be a strength of our curriculum by respondents). However, the major principles we incorporated into the design and implementation of this curriculum are generalizable to any setting where new staff need to be onboarded to the nuances of a dynamic clinical environment. These major principles include developing and disseminating a targeted needs assessment, choosing scenarios that match the needs assessment but can be flexible with changes in the clinical environment, and ensuring the engagement of leadership and new attending physicians in the process.

One unexpected benefit of the curriculum was social in nature. While not substantially reflected in the evaluation data, in casual conversation the participants expressed appreciation of the opportunity to work with and learn from their fellow new attending physicians in the psychologically safe space of simulation. After several of the simulation sessions, the participants scheduled more social time together away from the emergency department. It seems that the simulation orientation curriculum also contributes to the development of a community of practice among the new attending physicians.

One challenge we experienced involved ensuring that our scenario choices and timing continued to be appropriate for the new hires each year. Tailoring the curriculum to the needs of new attending physicians is an on‐going challenge as new attending physicians that have previously completed training at our institution may have very different needs for orientation compared to new attending physicians who are entirely new to the institution. As each year's composition of new attending physicians is different, the contents of the curriculum will need to be periodically adjusted to ensure maximal utility for the learners. Similarly, the clinical context in which the new attending physicians will be practicing is continuously evolving and therefore the scenarios will need to reflect those changes as well. For example, in the first year of our curriculum, we chose status epilepticus that required transfer to the main campus as a topic for the session at the satellite hospital ED, but by the time the second year of the curriculum occurred, a neurology service was available at the satellite hospital which obviated the need to transfer and thus the discussion of the logistics of transfer. As practicing the logistics of an emergency inter‐facility transport was one of our main learning objectives, we subsequently changed the scenario to ventriculoperitoneal shunt failure to force the participants to simulate the process of transferring a very sick patient to our main campus to be evaluated by the neurosurgery team, which does not cover the satellite hospital.

Looking forward, we would like to better understand the objective impact our orientation simulation curriculum has on new attending physicians' exposure to acute resuscitations or clinical scenarios in our ED. Due to the relative rarity of pediatric resuscitations and the large size of our overall faculty (over 70 attending physicians at the inception of the curriculum), any individual new faculty's experience in the resuscitation bay will be infrequent, limiting the ability to determine the effect of our curriculum on their performance rather than maturation effect [24]. Despite these challenges, we believe that we have developed an effective method of orienting new attending physicians to our emergency department, and we would strongly advocate for the inclusion of simulation into the development of any new attending physician orientation curriculum.

## Author Contributions

M.H., M.F., G.G., T.W., K.‐Y.T., and M.L. contributed to study concept and design. M.H. contributed to acquisition of the data. M.H. and K.‐Y.T. contributed to analysis and interpretation of the data. M.H., M.F., G.G., T.W., K.‐Y.T., and M.L. contributed to drafting of the manuscript. K.‐Y.T., T.W., and M.L. contributed to critical revision of the manuscript for important intellectual content.

## Funding

The authors have nothing to report.

## Conflicts of Interest

The authors declare no conflicts of interest.

## Supporting information


**Appendix S1:** Needs assessment.

## Data Availability

The data that support the findings of this study are available from the corresponding author upon reasonable request.
