# Supplementary material for: The Development and Implementation of a Simulation Orientation Curriculum for Newly Hired Pediatric Emergency Medicine Attending Physicians
Source: AEM Educ Train. 2026 Feb 18;10(1):e70138. doi: 10.1002/aet2.70138 (PMC12916445; doi:10.1002/aet2.70138)
Supplement: Supplementary file 1 — Appendix S1: Needs assessment. [file AET2-10-e70138-s001.pdf]

New Faculty Simulation Curriculum Needs Assessment

We are developing a yearlong simulation-based curriculum for new PEM faculty to:

a) introduce systems-based nuances (a.k.a. “CHOPisms”) specific to leading resuscitations

b) continue to foster development of specific leadership skills and teamwork principles frequently utilized in the bay with our interprofessional and multidisciplinary colleagues

The goal of this survey is to:

a) assess interest in a longitudinal curriculum aimed at new faculty in developing skills in resuscitation leadership and crisis resource management as it applies to the CHOP emergency department clinical spaces

b) identify the clinical scenarios to be prioritized in developing this longitudinal curriculum for new faculty.

Your answers will be anonymous, so please feel free to be honest and candid in your answers.

How prepared did you feel clinically for your first experience leading a resuscitation in the CHOP ED resuscitation bay?	◯ Prepared<br>◯ Somewhat Prepared<br>◯ Neutral<br>◯ Somewhat Unprepared<br>◯ Unprepared
Please describe why you feel you were prepared to be in this situation	__________________________________
Please describe why you feel you were somewhat prepared to be in this situation	__________________________________
Please explain your answer	__________________________________
Please describe why you feel you were somewhat unprepared to be in this situation	__________________________________
Please describe why you feel you were unprepared to be in this situation	__________________________________
How prepared did you feel to handle the logistics for your first experience leading a resuscitation in the CHOP ED resuscitation bay (ex. calling a tier, activating massive transfusion protocol, getting consultants)?	◯ Prepared<br>◯ Somewhat Prepared<br>◯ Neutral<br>◯ Somewhat Unprepared<br>◯ Unprepared
Please describe why you feel you were prepared to be in this situation	__________________________________
Please describe why you feel you were somewhat prepared to be in this situation	__________________________________

Please explain your answer

____

Please describe why you feel you were somewhat unprepared to be in this situation

____

Please describe why you feel you were unprepared to be in this situation

____

Please mark your level of agreement with this statement:

Simulation training for CHOP ED resuscitation scenarios would have been helpful during my first year as an attending.

- ◯ Strongly Agree
- ◯ Agree
- ◯ Neutral
- ◯ Disagree
- ◯ Strongly Disagree

Please provide an explanation, details or examples why you chose this level of agreement:

____

Please mark your level of agreement with this statement:

I would have benefitted from a simulation curriculum if it had been offered in my first year as an attending

- ◯ Strongly Agree
- ◯ Agree
- ◯ Neutral
- ◯ Disagree
- ◯ Strongly Disagree

Please provide an explanation, details or examples why you chose this level of agreement:

____

Please consider the following clinical simulation scenarios and choose the most appropriate placement for the scenario within this yearlong curriculum. We may combine some of these scenarios into one simulation, but please try to consider each scenario separately. Please only pick "Early, within first 3 months of starting" for FOUR scenarios.

	Early, within first 3 months of starting (only pick 4)	Anytime within first 6 months	Anytime within first year	Do not think this scenario should be in the resuscitation simulation curriculum for new faculty
Septic shock with activation of PICU Tier system	○	○	○	○
Level 1 Trauma Alert	○	○	○	○
Cardiac Arrest with non-shockable rhythm (non-SIDS)	○	○	○	○
Cardiac Arrest with shockable rhythm	○	○	○	○
Non-cardiac ECMO activation	○	○	○	○
Cardiac ECMO activation	○	○	○	○
Critical status asthmaticus	○	○	○	○
Deteriorating patient with bronchiolitis on NIPPV	○	○	○	○
Difficult Airway Team activation	○	○	○	○
Cardioversion (e.g. unstable SVT or VT with pulse)	○	○	○	○
SIDS without ROSC/End of Life Care	○	○	○	○
Status epilepticus	○	○	○	○
Agitated teenager requiring restraints and IM medications	○	○	○	○

Are there any other specific scenarios that you think would be beneficial for new attendings to experience in their first year of becoming an attending faculty that are not listed above? ____________________________________

Please comment why you thought we should start with the scenarios below:

Septic shock with activation of PICU Tier system

____

Level 1 Trauma Alert

____

Cardiac Arrest with non-shockable rhythm (non-SIDS)

____

Cardiac Arrest with shockable rhythm

____

Non-cardiac ECMO activation

____

Cardiac ECMO activation

____

Critical status asthmaticus

____

Deteriorating patient with bronchiolitis on NIPPV

____

Difficult Airway Team activation

____

Cardioversion (e.g. unstable SVT or VT with pulse)

____

SIDS without ROSC/End of Life Care

____

Status epilepticus

____

Agitated teenager requiring restraints and IM medications

____

Please let us know the appropriate balance of actual scenario setting that would best for meeting the above learning goals:

All at CHOP Philadelphia — Evenly Split — All at CHOP King of Prussia

(Place a mark on the scale above)

Comment on scenario hospital setting choice:

____

Did you do your PEM fellowship training at CHOP?

◯ Yes
◯ No
